# Event and Comment

**Published:** 1913-12-15

**Authors:** 


### DENTAL REGISTER.

**Vol. LXVII. December 15, 1913. No. 12**

### EVENT AND COMMENT.

THE DENTAL RELIEF FUND. The committee appointed by the National Association to organize and conduct a campaign for raising a respectable fund to assist unfortunate and aged practitioners evidently means business, as will be evidenced by reading two communications in this issue from Doctors Gaylord and McManus. We hope all our readers will carefully consider this proposition as it has real merit in itself and deserves our cordial support. The men who are managing the campaign are all big hearted and devoted practitioners of years, and their minds are evidently set on accomplishing something worth while. The fact that they have secured over seven hundred dollars in a brief campaign in one state only ought to make us all feel that it is up to us to fall into line and help. What a fine thing it would be if the 20,000 ethical practitioners of our country could send a five dollar bill to the committee as a Christmas offering, and get five hundred of their seals that we could use to let the public know the dental profession is conscious of its duty to its own and is determined to care for them in a substantial manner. If we could only present this matter to the profession in some manner that would bring out an instant and spontaneous response, how good we should all feel, and what a lot of comfort and good cheer this would bring to many an anxious one. Theoretically it could be done without any one ever feeling worse for the gift, but practically we know that there are so many who will make excuses and try to convince themselves that it is a matter that does not concern or interest them and so they will do nothing. There should be at least 10,000 dentists out of 40,000 in this country who could and would be persuaded to contribute five dollars to this fund if the matter could be put up to them in its true light. We trust the committee in charge will keep up the good work and that every one that can will lend a hand.

ANOTHER YEAR. We have just finished making up the index for the sixty-seventh volume of the REGISTER, and we are impressed with the record of things done and attempted as we have recorded them. We cannot boast of any great accomplishment personally, but we feel that our profession has made substantial progress, and we have tried to make such record of it as was practicable. If one could take up the bound volumes of all the Dental Journals for 1913 and read them through he would probably realize that we were doing something in a literary way. When we realize the fact that about fifteen journals printed regularly in this country, supply us with about ten thousand pages of reading matter during the year, we get some idea of what one would have to do to keep up with our magazine literature alone, to say nothing of the foreign journals and books that are issued during the year. If this material was all printed in one book it would make a volume twice the size of the standard or Webster's Dictionary. Fortunately we don't have to read it all, as much of it is reprinted material, announcements, and many of the articles don't interest a large number of dentists. Some one has said that when the new journal of the National Association is started that the other journals will quit business. It certainly will be some journal that will take all this matter, and it will have to be printed weekly or probably daily to meet the demands made on it. As we look at it there is plenty of room for the new journal and we trust it will fulfill all the expectations of its most ardent supporters; if it shall raise the standard of our literary output it certainly will meet with a cordial welcome from every true professional man as well as every dental journal now in existence. Our dental journals are after all merely the score boards on which we mark up month by month the progress of the game by innings, as we play it. The superficial observer thinks the game is played out and he quits watching the score-board, but as is often the case the most exciting and profitable feature is pulled off in the next inning. Who can say what will happen this next year? We shall need our score-boards and we can't keep our eyes closed to them.

TROUBLES OF AN EXAMINING BOARD. From the daily newspaper we learn that a serious charge has been made against a member of one of the neighboring states Dental Examining Board. It seems that a new Governor took a notion to throw over the old examining board and appointed an entirely new one of his own political faith. The new Board had hardly got organized when one of its members is accused of selling, through a second party, his examination questions for fifty dollars. The matter is creating a good deal of excitement and if the charge is substantiated it will undoubtedly result in a strenuous effort to devise some way of controlling the appointment of our dental examiners other than by political influence. We are hoping when the matter is finally investigated that the charge may not be sustained. At any rate it does seem strange that we can't have men that are above suspicion in all places of trust. Political preferment seems to have a mighty influence in attracting the morally weak and unfit. When we get to that high plane of thinking and living on which every man who accepts a preferment of any sort will feel that he is called to a sacred trust that he must never betray, we shall have unselfish and satisfactory service. Until then we shall have to put up with the frailties of men and the shortcomings of our standards of living and government.

